# Transcriptome Signatures Reveal Rapid Induction of Immune-Responsive Genes in Human Memory CD8^+^ T Cells

**DOI:** 10.1038/srep27005

**Published:** 2016-05-31

**Authors:** Cheng Yang, Asma Khanniche, Joanna R. DiSpirito, Ping Ji, Shujun Wang, Ying Wang, Hao Shen

**Affiliations:** 1 Shanghai Institute of Immunology, Shanghai Jiaotong University School of Medicine, Shanghai, China; 2 Department of Microbiology, University of Pennsylvania Perelman School of Medicine, Philadelphia, PA, USA

## Abstract

Memory T cells (T_M_) play a prominent role in protection and auto-immunity due to their ability to mount a more effective response than naïve T cells (T_N_). However, the molecular mechanisms underlying enhanced functionality of T_M_ are not well defined, particularly in human T_M_. We examined the global gene expression profiles of human CD8^+^ T_N_ and T_M_ before and after stimulation. There were 1,284, 1,373 and 1,629 differentially expressed genes between T_N_ and T_M_ at 0 hr, 4 hr and 24 hr after stimulation, respectively, with more genes expressed to higher levels in T_M_. Genes rapidly up-regulated in T_N_ cells were largely involved in nitrogen, nucleoside and amino acid metabolisms. In contrast, those in CD8^+^ T_M_ were significantly enriched for immune-response-associated processes, including cytokine production, lymphocyte activation and chemotaxis. Multiple cytokines were rapidly up-regulated in T_M_ cells, including effector cytokines known to be produced by CD8^+^ T cells and important for their functions, as well as regulatory cytokines, both pro- and anti-inflammatory, that are not typically produced by CD8^+^ T cells. These results provide new insights into molecular mechanisms that contribute to the enhanced functionality of human CD8^+^ T_M_ and their prominent role in protection and auto-immunity.

The peripheral CD8^+^ T cell repertoire is highly heterogeneous, consisting of naïve (T_N_), effector (T_E_) and memory (T_M_) cell subsets. CD8^+^ T_N_ cells become activated and give rise to T_E_ cells after priming by exogenous antigens, while T_E_ cells differentiate into T_M_ cells following the withdrawal of antigenic and inflammatory stimulation^1^,^2^. CD8^+^ T_M_ cells display the unique property of rapid recall responses, characterized by immediate effector cytokine production and rapid proliferation upon antigen re-encounter^1^,^3^,^4^,^5^. These characteristics of CD8^+^ T_M_ cells have been mostly defined in murine infection models, where infection can be controlled and antigen-specific T cells can be tracked by using TCR-transgenic cells^6^,^7^,^8^,^9^. However, far less is known for human T_M_ cells, where the combination of natural infections and vaccinations re-occur over a lifetime, likely yielding a highly heterogeneous population of T_M_ cells that are continually alternating between a resting and stimulated state.

Over the past two decades, efforts have been made to uncover the molecular basis for the functionality of human T cells. Indeed, human CD8^+^ T cells expressing memory-cell-identifying surface markers (such as CD45RO) have been found to be able to respond rapidly to stimulation^10^. Using gene expression microarrays, a number of studies have examined global transcriptional profiles in human CD8^+^ T_N_ and T_M_ at the resting state, and have identified transcriptional signatures that correlate with their homeostasis and functionality^11^,^12^,^13^. However, less is known about the early, stimulation-induced transcriptional programming of human and even murine T_M_ cells. While studies of gene expression in quiescent T_M_ cells can reveal mechanisms underlying their homeostasis and maintenance, it is the genes rapidly induced after stimulation that participate in the execution of immune functions *in vivo*, and likely play a prominent role in contributing to the enhanced functionality of CD8^+^ T_M_ cells.

In this study, we compared global gene expression profiling of human CD8^+^ T_N_ and T_M_ cells before and shortly after stimulation. We have identified a cluster of immune-response-associated genes that were rapidly induced after stimulation in CD8^+^ T_M_ cells but not in their T_N_ counterparts. Our results thus provide new insights into the enhanced functionality of human CD8^+^ T_M_ cells.

## Results

### Rapid changes in global gene expression in both CD8^+^ T_N_ and T_M_ following stimulation

**Figure 1:**
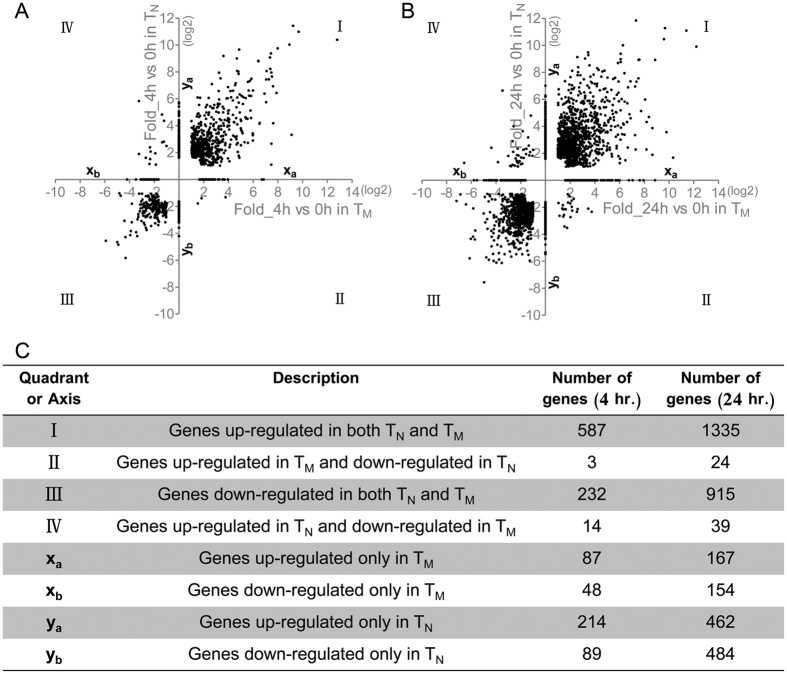
Stimulation-induced gene expression changes in CD8^+^ T_N_ and T_M_ cells. CD8^+^ T_M_ and T_N_ were purified from two individuals and stimulated with anti-CD3/CD28 for 0, 4 or 24 hr, and genome-wide expression profiles were determined by microarray. Individual genes were plotted by their fold up-regulation or down-regulation following 4 hrs’ (**A**) or 24 hrs’ (**B**) stimulation in comparison with the resting state. Genes differentially expressed in both cell types were clustered into quadrants, and those differentially expressed only in one cell type were plotted on axes. The table (**C**) listed the number of genes falling into each cluster.

To uncover molecular mechanisms underlying the enhanced functionality of human CD8^+^ T_M_ cells, we stimulated purified CD8^+^ T_N_ (CD8^+^CD4^−^CD45RO^−^CD27^+^) and T_M_ (CD8^+^CD4^−^CD45RO^+^) cells with anti-CD3/CD28 for 0, 4, and 24 hr, respectively, and measured mRNA transcripts on genome-wide microarrays. There were 1,274 genes differentially expressed, either up- or down-regulated, at 4 hr post-stimulation (Fig. 1A). Most of them were commonly induced (cluster I, 587 genes) or repressed (cluster III, 232 genes) in both T_M_ and T_N_ cells. Three genes showed “opposite” changes, which were up-regulated in T_M_ cells while down-regulated in T_N_ cells (cluster II), or contrarily, fourteen genes were up-regulated in T_N_ cells while down-regulated in T_M_ cells (cluster IV). Finally, more genes were uniquely induced (cluster y_a_ vs. x_a_, 214 genes for CD8^+^ T_N_ vs. 87 genes for CD8^+^ T_M_) or suppressed (cluster y_b_ vs. x_b_, 89 genes for CD8^+^ T_N_ vs. 48 genes for CD8^+^ T_M_) in CD8^+^ T_N_ than in T_M_ cells (Fig. 1C). Similar expression profiles were observed at 24 hr post stimulation with more differential genes detectable in both CD8^+^ T_M_ and T_N_ cells compared to 4 hr time point (Fig. 1B,C). Thus, stimulation induces changes in expression of a large number of genes in both CD8^+^ T cell subsets.

### Distinct transcriptional signatures in CD8^+^ T_M_ and T_N_ cells

**Figure 2:**
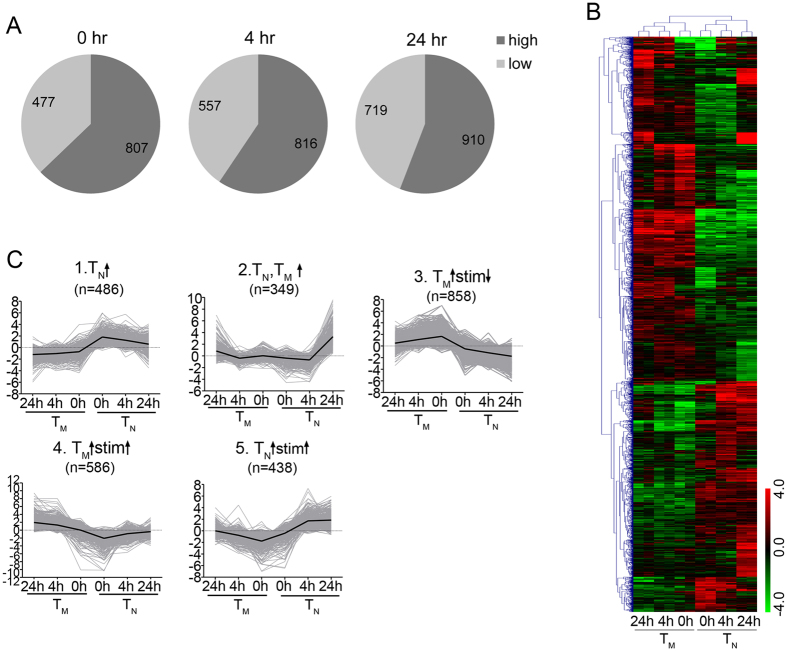
Different transcriptional signatures between CD8^+^ T_M_ and T_N_ cells at resting and after stimulation. (**A**) Numbers of differential genes between CD8^+^ T_M_ and T_N_ at 0 hr, 4 hr and 24 hr after stimulation. High (low), genes expressed to higher (lower) levels in CD8^+^ T_M_ than in T_N_ cells. (**B**) Hierarchical clustering analysis of genes differentially expressed between CD8^+^ T_M_ and T_N_ at resting or 4 hr, 24 hr after stimulation (duplicate samples). (**C**) K-means clustering of differentially expressed genes. Gray curves showed the transcription profiles of individual genes, with a heavy curve indicating the overall pattern in each cluster. ↑, increased or induced; ↓, decreased or repressed; stim, anti-CD3/CD28 stimulation.

We next compared the transcriptional signatures between CD8^+^ T_M_ and T_N_ cells both at the resting state and after stimulation. There were 1,284 differential genes between T_N_ and T_M_ cells prior to stimulation (0 hr), with 807 of them expressed to higher levels in CD8^+^ T_M_ cells (Fig. 2A and Supplementary Table S2). At 4 hr post activation, the number of differential genes increased slightly to 1,373, with 816 of them expressed to higher levels in CD8^+^ T_M_ cells (Fig. 2A and Supplementary Table S2). At 24 hr after activation, we found the highest number of differential genes (1,629) with 910 of them more highly expressed in T_M_ cells (Fig. 2A and Supplementary Table S2). These results indicate that more genes are expressed to higher levels in CD8^+^ T_M_ than in T_N_ shortly following stimulation.

To give an overall view of differential genes, we performed hierarchical clustering analysis on all genes that were differentially expressed between CD8^+^ T_M_ and T_N_ at 0, 4, or 24 hr post stimulation (Fig. 2B). Next, using K-means clustering, we identified five major modules of genes with similar expression patterns: 1) consistently expressed more highly in T_N_ than in T_M_; 2) inducible in both T_N_ and T_M_ after stimulation; 3) initially expressed highly in T_M_ and suppressed after stimulation in both T_N_ and T_M_; 4) consistently expressed to higher levels in CD8^+^ T_M_ and further up-regulated by stimulation; and 5) highly expressed in CD8^+^ T_N_ and further up-regulated by stimulation (Fig. 2C). Together, these clustering analyses reveal that CD8^+^ T_M_ and T_N_ cells display distinct transcriptional signatures both at the steady state and after stimulation.

### Rapid up-regulation of genes associated with immune response processes in human CD8^+^ T_M_ cells upon stimulation

**Figure 3:**
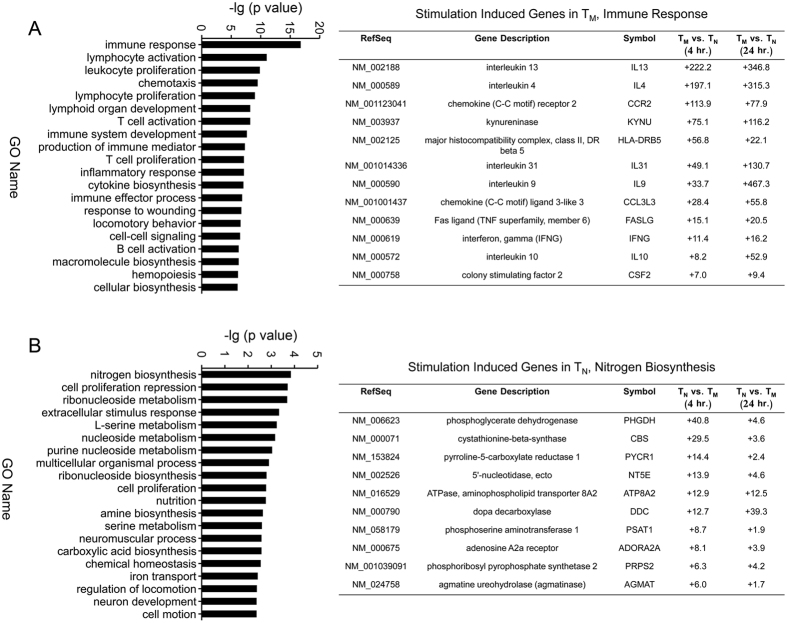
Functional enrichment assay for genes robustly induced in CD8^+^ T_M_ and T_N_ cells upon stimulation. (**A**) Gene Ontology analysis of genes highly expressed and induced by stimulation in CD8^+^ T_M_ (Cluster 4 in Fig. 2C) (left) with a table (right) showing individual highly induced genes in CD8^+^ T_M_ cells that are associated with immune-response processes. (**B**) Gene Ontology analysis of genes highly expressed and induced by stimulation in CD8^+^ T_N_ (Cluster 5 in Fig. 2C) (left). The table (right) shows individual highly induced genes in T_N_ associated with nitrogen biosynthesis.

Functional enrichment analysis of differentially expressed genes could shed light on their roles in the rapid recall program of CD8^+^ T_M_ cells. Thus, we performed gene ontology (GO) analysis for differentially expressed genes. Genes that were initially highly expressed and induced upon stimulation only in CD8^+^ T_M_ (Cluster 4 in Fig. 2C) were most likely to be involved in rapid responses. We thus examined the functional enrichment for this cluster of genes. Our analysis revealed that this cluster was significantly enriched (p < 0.05) in multiple key immune processes, including immune response, chemotaxis, cytokine production, T cell activation and proliferation, all of which are crucial processes for the effector functions of CD8^+^ T_M_ cells (Fig. 3A, left). Consistent with these results, many cytokine and chemokine genes were robustly induced by short-term stimulation in CD8^+^ T_M_ in this cluster (Fig. 3A, right). In contrast, genes highly expressed and induced by stimulation in CD8^+^ T_N_ (Cluster 5 in Fig. 2C) were mostly related to metabolism of nitrogen, nucleosides and amino acids (Fig. 3B). These data indicate that robustly induced genes in CD8^+^ T_M_ and T_N_ cells are enriched in different functional categories, with the former ones strongly enriched for immune-response-associated processes, suggesting that these genes might underline the enhanced functionality of CD8^+^ T_M_.

### Increased expression of pro- and anti-inflammatory cytokines by CD8^+^ T_M_ cells

**Figure 4:**
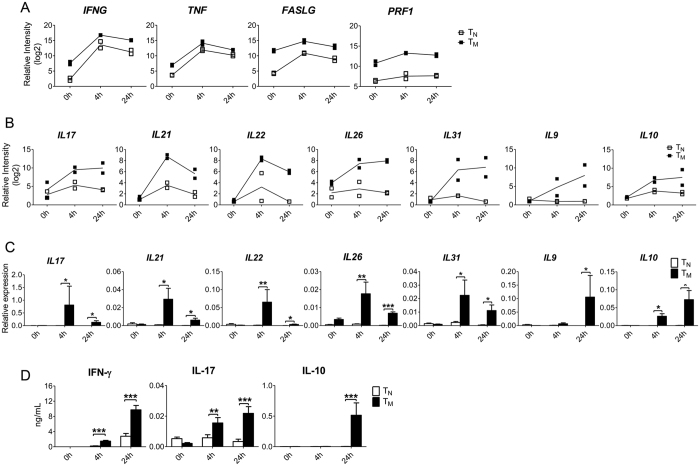
Enhanced expression of effector molecules and cytokines by CD8^+^ T_M_ cells. Purified CD8^+^ T_M_ and T_N_ cells were stimulated with anti-CD3/CD28 for 0 hr, 4 hr and 24 hr. (**A**) The mRNA levels of *IFNG*, *TNF*, *FASLG* and *PRF1* were determined by microarray analysis. (**B**,**C**) The mRNA levels of *IL17*, *IL21*, *IL22*, *IL26*, *IL31*, *IL9* and *IL10* were measured by microarray (**B**) and were validated by qRT-PCR (**C**). (**D**) The protein level of IFN-γ, IL-17 and IL-10 in the supernatants was measured by ELISA. Data in (**C**,**D**) were from 8 independent experiments. p value is indicated by asterisks (*p < 0.05, **p < 0.01, ***p < 0.001).

Many cytokines are among genes that were rapidly up-regulated to high levels after stimulation only in CD8^+^ T_M_ (Cluster 4 in Fig. 2C). Cytokines play important roles in host immune responses and can have effector or regulatory functions that are either pro- or anti-inflammatory. We further examined which cytokines were rapidly induced to high levels in CD8^+^ T_M_ but not in T_N_. As expected^12^,^14^, they included typical effector molecules of CD8^+^ T cells, such as *IFNG*, *TNF*, *PRF1* and *FASLG* (Fig. 4A). Interestingly, we also found in this cluster pro- and anti-inflammatory cytokines that are not commonly produced by CD8^+^ T cells, including *IL17*, *IL21*, *IL22*, *IL26*, *IL31*, *IL9* and *IL10* (Fig. 4B and Supplementary Table S2). For a more quantitative analysis, we purified CD8^+^ T_M_ and T_N_ cells independent from those used for microarrays, and stimulated both cell types with anti-CD3/CD28 beads for 0, 4 and 24 hr. Expression of these genes was quantified at mRNA levels by quantitative RT-PCR. Consistent with our microarray data, all seven cytokine genes were expressed to higher levels in CD8^+^ T_M_ either at 4 hr or 24 hr after stimulation (Fig. 4C). Furthermore, we measured the protein levels of key cytokines, including IFN-γ, IL-17, and IL-10. Consistent with the microarray and qRT-PCR data, we found that levels of these cytokines were much higher in supernatants of CD8^+^ T_M_ cultures than in supernatants of T_N_ after stimulation (Fig. 4D). Therefore, an expanded cytokine spectrum is observed in human CD8^+^ T_M_ cells following stimulation, which might contributes to the enhanced functionality observed in CD8^+^ T_M_ cells.

## Discussion

Immune responses are composed of heterogeneous collections of cell types with diverse functional capacities. Among T cells, memory subsets are longer-lived than their effector counterparts and are more potent responders on a per-cell basis than their naïve precursors. When stimulated, T_M_ cells enter a state of clonal expansion and effector molecule production more rapidly than T_N_ cells^15^. Thus, eliciting T_M_ cells that are highly functional and persist over time is a goal of vaccination strategies^16^. Conversely, inhibition of the robust recall ability of T_M_ cells is desirable in clinical treatments for autoimmune diseases and transplantation tolerance^17^,^18^. A detailed characterization of human T_M_ cells’ behavior following early stimulation, which is when they are the most functional, will inform the development of therapeutic strategies for eliciting highly functional T_M_ cells, or for restraining pathologic ones. We approached this problem by utilizing whole genome expression profiling to capture the stimulation-responsive transcriptional signatures in human CD8^+^ T_M_ cells. To identify key molecular players in stimulated CD8^+^ T_M_ cells, we compared this program to the one that occurs following stimulation of their naïve T cell counterparts. Stimulation induced changes in expression of a large number of genes in both CD8^+^ T_N_ and T_M_, with many of them commonly induced (587 genes) or repressed (232 genes) in both T_N_ and T_M_. However, there were also many genes that were differentially expressed between CD8^+^ T_M_ and T_N_ cells shortly after stimulation (1,373 at 4 hr and 1,629 at 24 hr, Fig. 2A,B). This is in contrast to only about a hundred genes that are differentially expressed between T_N_ and T_M_ at resting state^11^,^12^,^13^. Functional enrichment analysis of differentially expressed genes at rest and after stimulation offers distinct insights into the unique features and functions of T_M_. At the resting state, a prominent group of genes that are highly expressed in T_M_ than in T_N_ are those involved in homing and homeostasis, and these factors are likely important in supporting the unique circulation pattern and long-term survival of T_M_ cells^19^. After stimulation, our results show that many genes encoding immune effector functions are rapidly induced to high levels in T_M_ compared to T_N_. These results are consistent with previous studies^12^,^14^, and provide a transcriptional basis for the enhanced functionality of T_M_. Importantly, our studies reveal rapid induction in human CD8^+^ T_M_ of many immune effectors/cytokines that are not commonly known to be expressed by CD8^+^ T cells, suggesting that these factors may play a role in contributing to the overall quality and functional diversity of human CD8^+^ T_M_ responses.

Among genes highly expressed and rapidly induced after stimulation in CD8^+^ T_M_ cells are many conventional type 1 effector cytokines, such as *TNF*, *IFNG* and *PRF1*. Production of these effector cytokines by T cells is clinically relevant and the key indicators for cell functionality, especially for memory CD8^+^ T cell responses^20^. For example, TNF-α and IFN-γ producing CD8^+^ T_M_ cells are associated with the control of intracellular pathogens on the one hand and with pathologic responses to self-tissues on the other^21^,^22^,^23^. We were surprised to find that type 2 cytokines, including *IL13*, *IL4* and *IL5*, were induced in CD8^+^ T_M_. While CD8^+^ T cells typically produce type 1 effector cytokines, production of type 2 cytokines by human CD8^+^ T_M_ has also been observed by Fann *et al.*^12^. Furthermore, CD8^+^ T cells can synthesize type 2 cytokines when cultured with IL-4 *in vitro*^24^, and *in vivo*-activated CD8^+^ type 2 cytokine producers exist in mice^25^. We also found that CD8^+^ T_M_ cells expressed high levels of Th17 associated cytokines (*IL17* and *IL22*), as well as many pro- and anti-inflammatory cytokines (such as *IL21*, *IL26*, *IL31*, *IL9* and *IL10*) shortly after stimulation. These cytokines are mainly produced by CD4^+^ T helper cells and many of them function not only as direct effectors but also as regulatory cytokines that control inflammatory responses. Specifically, IL-17 derived from Th17 cells is involved in the pathogenesis of inflammatory and autoimmune diseases, such as multiple sclerosis (MS) and rheumatoid arthrosis (RA)^26^,^27^. IL-21 is the key effector cytokine of the recently described T follicular helper cells (Tfh)^28^ and IL-9 producing Th9 cells contribute to a wide range of inflammatory and allergic diseases in mice model^29^. In addition, IL-10 is responsible for preventing excessive inflammation^30^. Here we show that human CD8^+^ T_M_ cells are also a source of these cytokines, raising the possibility that CD8^+^ T_M_ cells may play a regulatory role in both protective and pathological circumstance. Expression of these various cytokines, including type 2 cytokines, likely reflects the complexity of how memory T cell populations in humans are generated, through successive encounters with numerous natural infections and vaccinations that drive different types of immune responses. The functional plasticity of human CD8^+^ T_M_ cell populations as a whole may allow human CD8^+^ T_M_ cells to quickly mount an appropriate, protective cytokine response to different re-infections. In addition to heterogeneity in cytokine production, the T_M_ population is composed of distinct subsets, including central, effector, and tissue-resident T_M_ that provide protection at different times and locations. At the resting stage, most of the highly expressed genes in CD8^+^ T_M_ are shared between central and effector T_M_, though there are differences between the two subsets^11^. Future studies analyzing the stimulation-induced transcriptomes of these T_M_ subsets will likely provide more insights into the responsiveness of the complex human T_M_ population.

Previous studies have focused on genes that are highly expressed and/or induced in human CD8^+^ T_M_ cells, as these genes certainly are major determinants of T_M_ functions^12^,^19^. Our comparative analysis of gene expression in CD8^+^ T_M_ and T_N_ after stimulation reveals five clusters of expression patterns (Fig. 2C). Genes consistently expressed to higher levels in T_N_ (cluster 1) were enriched in cell maturation and biological adhesion processes. Those induced by stimulation in both T_N_ and T_M_ (cluster 2) were enriched in cell cycle, M phase and nuclear division, which are probably responsible for the rapid proliferation of both cell types upon stimulation. Genes consistently expressed higher in T_M_ (in cluster 3) were mostly involved in immune response and signal transduction (data not shown). The stimulation-responsive genes in CD8^+^ T_M_ were mostly associated with immunological pathways, including cytokine secretion, and lymphocyte proliferation (cluster 4). In contrast, genes differentially expressed after stimulation in CD8^+^ T_N_ cells largely included metabolic regulation genes with functions in DNA replication and cell proliferation rather than immune responses (cluster 5). Future studies of differentially expressed genes in all clusters, not simply those highly expressed/induced in T_M_, will help us gain a complete understanding of T_M_ differentiation, maintenance and functionality.

Taken together, our transcriptional signature data presented here show that a large set of immune-responsive genes are rapidly induced in human CD8^+^ T_M_ cells upon stimulation. The stimulation-responsive genes in CD8^+^ T_M_ include effector cytokines known to be produced by, and important for CD8^+^ T cell function. In addition, we found many regulatory cytokines, both pro- and anti-inflammatory, were rapidly induced to high levels in CD8^+^ T_M_. These results provide new insights into molecular mechanisms that contribute to the enhanced functionality of human CD8^+^ T_M_ and their prominent roles in protection and auto-immunity.

## Methods

### Isolation and stimulation of CD8^+^ T cells

Peripheral blood mononuclear cells (PBMCs) from healthy donors were isolated by Ficoll gradient centrifugation with Lymphoprep^TM^ solution (AXIS-SHIELD Poc AS). CD8^+^ T cells were enriched using human CD8^+^ T cell isolation kit (Miltenyi Biotec.) with purity always >90% achieved. CD8^+^ T_M_ and T_N_ cells were further sorted on FACS Aria II (BD Biosciences) based on the surface expression of CD27 and CD45RO, with purity greater than 98% obtained each time. Freshly sorted CD8^+^ T_M_ and T_N_ cells were cultured in R10 medium (RPMI 1640 + 10% fetal bovine serum + 100 U/mL Penicillin/Streptomycin) (GIBCO) at a cell concentration of 2 × 10^6^/mL in 96-U-bottom plates, and stimulated with anti-CD3 and anti-CD28 mAbs coupled magnetic beads (Invitrogen) at a cell-bead ratio of 1:1 for 4 hr, 24 hr or left unstimulated. Cells and culture supernatants were harvested after stimulation.

### Microarray analysis

Total RNA from CD8^+^ T_M_ and T_N_ cells was purified using miRNeasy Kits (Qiagen) and RNA integrity was analyzed by Agilent Bioanalyzer 2100 (Agilent technologies). Microarray experiments were performed with whole-human-genome 4*44K arrays (Agilent) on duplicate samples by Shanghai SBS Company (Shanghai) following the manufacture’s standard protocols. Array data were normalized by Gene Spring Software 11.0 (Agilent), and normalized data were used for calculating fold changes in expression. Those with >3-fold in both individuals were considered as differentially expressed. Heatmap of differential genes was generated with MultiExperiment Viewer software (MeV). Functional annotation of genes of interests was carried out with DAVID Bioinformatics Resources (http://david.a^®^bcc.ncifcrf.gov/home.jsp). The complete data sets of gene expression profile in resting and stimulated CD8^+^ T cell subsets can be found at the NCBI Gene Expression Omnibus with accession number GSE79828.

### Quantitative PCR

Total RNA was extracted by using Trizol Reagent (Invitrogen) and cDNA was synthesized with PrimeScript RT reagents (Takara Bio Inc.). Quantification of gene expression levels was performed on a 7500 Fast Real-Time PCR cycler (Applied Biosystems) with SYBR^®^ Green reagents (Takara Bio Inc.). Primers of specific genes were designed and synthesized by BioTNT co. (Shanghai, China) and their sequences have been listed in Supplementary Table S1. Gene expression levels were normalized to *GAPDH* according to the 2^−ΔCt^ method.

### ELISA

Cytokines in culture supernatants were determined by using Cytokine Detection kits (R&D Systems Inc.) according to the manufacturer’s instructions.

### Statistical analysis

The data were represented by mean ± S.E.M. Statistical difference was determined by Student two-tailed *t* test using GraphPad Prism software (GraphPad, San Diego, CA). *p* value < 0.05 was considered statistically significant.

### Study approval

This study was conducted according to the Declaration of Helsinki. All protocols were reviewed and approved by the ethical committee of Shanghai Jiaotong University, School of Medicine. Informed consent was obtained from all volunteers.

## Additional Information

**How to cite this article**: Yang, C. *et al.* Transcriptome Signatures Reveal Rapid Induction of Immune-Responsive Genes in Human Memory CD8^+^ T Cells. *Sci. Rep.* **6**, 27005; doi: 10.1038/srep27005 (2016).

## Supplementary Material

Supplementary Information

Supplementary Table S2

## References

[b1] Kaech S. M., Wherry E. J. & Ahmed R. Effector and memory T-cell differentiation: implications for vaccine development. Nature reviews. Immunology 2, 251–262, 10.1038/nri778 (2002). 12001996

[b2] Goldrath A. W., Luckey C. J., Park R., Benoist C. & Mathis D. The molecular program induced in T cells undergoing homeostatic proliferation. Proceedings of the National Academy of Sciences of the United States of America 101, 16885–16890, 10.1073/pnas.0407417101 (2004). 15548615 PMC534746

[b3] Harty J. T., Tvinnereim A. R. & White D. W. CD8^+^ T cell effector mechanisms in resistance to infection. Annual review of immunology 18, 275–308, 10.1146/annurev.immunol.18.1.275 (2000). 10837060

[b4] Williams M. A. & Bevan M. J. Effector and memory CTL differentiation. Annual review of immunology 25, 171–192, 10.1146/annurev.immunol.25.022106.141548 (2007). 17129182

[b5] Gourley T. S., Wherry E. J., Masopust D. & Ahmed R. Generation and maintenance of immunological memory. Seminars in immunology 16, 323–333, 10.1016/j.smim.2004.08.013 (2004). 15528077

[b6] Kaech S. M., Hemby S., Kersh E. & Ahmed R. Molecular and functional profiling of memory CD8 T cell differentiation. Cell 111, 837–851 (2002). 12526810 10.1016/s0092-8674(02)01139-x

[b7] Wherry E. J. & Ahmed R. Memory CD8 T-cell differentiation during viral infection. Journal of virology 78, 5535–5545, 10.1128/JVI.78.11.5535-5545.2004 (2004). 15140950 PMC415833

[b8] Shedlock D. J. & Shen H. Requirement for CD4 T cell help in generating functional CD8 T cell memory. Science 300, 337–339, 10.1126/science.1082305 (2003). 12690201

[b9] Kaech S. M. & Ahmed R. Memory CD8^+^ T cell differentiation: initial antigen encounter triggers a developmental program in naive cells. Nature immunology 2, 415–422, 10.1038/87720 (2001). 11323695 PMC3760150

[b10] Hamann D. *et al.* Phenotypic and functional separation of memory and effector human CD8^+^ T cells. The Journal of experimental medicine 186, 1407–1418 (1997). 9348298 10.1084/jem.186.9.1407 PMC2199103

[b11] Willinger T., Freeman T., Hasegawa H., McMichael A. J. & Callan M. F. Molecular signatures distinguish human central memory from effector memory CD8 T cell subsets. Journal of immunology 175, 5895–5903 (2005). 10.4049/jimmunol.175.9.5895 16237082

[b12] Fann M. *et al.* Histone acetylation is associated with differential gene expression in the rapid and robust memory CD8(^+^) T-cell response. Blood 108, 3363–3370, 10.1182/blood-2006-02-005520 (2006). 16868257 PMC1895425

[b13] Holmes S., He M., Xu T. & Lee P. P. Memory T cells have gene expression patterns intermediate between naive and effector. Proc Natl Acad Sci USA 102, 5519–5523, 10.1073/pnas.0501437102 (2005). 15809420 PMC556264

[b14] Araki Y., Fann M., Wersto R. & Weng N. P. Histone acetylation facilitates rapid and robust memory CD8 T cell response through differential expression of effector molecules (eomesodermin and its targets: perforin and granzyme B). Journal of immunology 180, 8102–8108 (2008). 10.4049/jimmunol.180.12.8102 PMC2493419 18523274

[b15] DiSpirito J. R. & Shen H. Quick to remember, slow to forget: rapid recall responses of memory CD8^+^ T cells. Cell research 20, 13–23, 10.1038/cr.2009.140 (2010). 20029390

[b16] Pulendran B. & Ahmed R. Immunological mechanisms of vaccination. Nature immunology 12, 509–517 (2011). 21739679 10.1038/ni.2039 PMC3253344

[b17] Bhargava P. & Calabresi P. A. Novel therapies for memory cells in autoimmune diseases. Clin Exp Immunol 180, 353–360, 10.1111/cei.12602 (2015). 25682849 PMC4449764

[b18] Krupnick A. S. *et al.* Central memory CD8^+^ T lymphocytes mediate lung allograft acceptance. J Clin Invest 124, 1130–1143, 10.1172/JCI71359 (2014). 24569377 PMC3938255

[b19] Weng N. P., Araki Y. & Subedi K. The molecular basis of the memory T cell response: differential gene expression and its epigenetic regulation. Nature reviews. Immunology 12, 306–315, 10.1038/nri3173 (2012). PMC4686144 22421787

[b20] Seder R. A. *et al.* Protection against malaria by intravenous immunization with a nonreplicating sporozoite vaccine. Science 341, 1359–1365, 10.1126/science.1241800 (2013). 23929949

[b21] Wehrens E. J., Prakken B. J. & van Wijk F. T cells out of control–impaired immune regulation in the inflamed joint. Nat Rev Rheumatol 9, 34–42 (2013). 23390638 10.1038/nrrheum.2012.149

[b22] Ignatius Arokia Doss P. M., Roy A. P., Wang A., Anderson A. C. & Rangachari M. The Non-Obese Diabetic Mouse Strain as a Model to Study CD8(+) T Cell Function in Relapsing and Progressive Multiple Sclerosis. Front Immunol 6, 541, 10.3389/fimmu.2015.00541 (2015). 26557120 PMC4617102

[b23] Jasenosky L. D., Scriba T. J., Hanekom W. A. & Goldfeld A. E. T cells and adaptive immunity to Mycobacterium tuberculosis in humans. Immunological reviews 264, 74–87, 10.1111/imr.12274 (2015). 25703553

[b24] Kelso A. & Groves P. A single peripheral CD8^+^ T cell can give rise to progeny expressing type 1 and/or type 2 cytokine genes and can retain its multipotentiality through many cell divisions. Proceedings of the National Academy of Sciences of the United States of America 94, 8070–8075 (1997). 9223316 10.1073/pnas.94.15.8070 PMC21558

[b25] Coyle A. J. *et al.* Virus-specific CD8^+^ cells can switch to interleukin 5 production and induce airway eosinophilia. The Journal of experimental medicine 181, 1229–1233 (1995). 7869040 10.1084/jem.181.3.1229 PMC2191899

[b26] Rostami A. & Ciric B. Role of Th17 cells in the pathogenesis of CNS inflammatory demyelination. Journal of the neurological sciences 333, 76–87, 10.1016/j.jns.2013.03.002 (2013). 23578791 PMC3726569

[b27] Noack M. & Miossec P. Th17 and regulatory T cell balance in autoimmune and inflammatory diseases. Autoimmunity reviews 13, 668–677, 10.1016/j.autrev.2013.12.004 (2014). 24418308

[b28] Gharibi T. *et al.* Biological effects of IL-21 on different immune cells and its role in autoimmune diseases. Immunobiology 221, 357–367, 10.1016/j.imbio.2015.09.021 (2016). 26466984

[b29] Jabeen R. & Kaplan M. H. The symphony of the ninth: the development and function of Th9 cells. Current opinion in immunology 24, 303–307, 10.1016/j.coi.2012.02.001 (2012). 22365614 PMC3368035

[b30] O’Garra A., Barrat F. J., Castro A. G., Vicari A. & Hawrylowicz C. Strategies for use of IL-10 or its antagonists in human disease. Immunological reviews 223, 114–131, 10.1111/j.1600-065X.2008.00635.x (2008). 18613832

