# Supplementary material for: Transcriptome Signatures Reveal Rapid Induction of Immune-Responsive Genes in Human Memory CD8+ T Cells
Source: Sci Rep. 2016 May 31;6:27005. doi: 10.1038/srep27005 (PMC4886650; doi:10.1038/srep27005)
Supplement: Supplementary Information [file srep27005-s1.pdf]

Supplementary Information

Transcriptome Signatures Reveal Rapid Induction of Immune-Responsive Genes in Human Memory CD8$^+$ T Cells

Cheng Yang[1#], Asma Khanniche[1#], Joanna R. DiSpirito[2], Ping Ji[1], Shujun Wang[1], Ying Wang[1]* and Hao Shen[1, 2]*

[1.] Shanghai Institute of Immunology, Shanghai Jiaotong University School of Medicine, Shanghai, China

[2.] Department of Microbiology, University of Pennsylvania Perelman School of Medicine, Philadelphia, PA, USA

Supplementary Table S1. Primer sequences used for qPCR in this study

GeneSymbol	RefSeq	Forward primer	Reverse primer
IL17	NM_052872	CACGTAACATCGAGAGCCG	AGCCCAAGTTCCTACACTGG
IL21	NM_021803	GTGAAAACGAGACCAAG	CCAGTGTCCCCAAGAAGAT
IL22	NM_020525	AAGTGCTGTTCCCTCAATCTG	ATGTGCTTAGCCTGTTGCTG
IL26	NM_018402	GTGGGTTGCTGTTAGTCACTCT	GTCTTCTGGAATCGTTGCTT
IL31	NM_001014336	GATGATGTACAGAAAATAGTCGAGGAATT	CTTCTCTTCCTCCACATCTTTCAAA
IL9	NM_000590	GTGCCACTGCAGTGCTAATGT	CTCTCACTGAAGCATGGTCTGG
IL10	NM_000572	GCCTAACATGCTTCGAGATC	TGATGTCTGGGTCTTGGTTC
GAPDH	NM_002046	GAAGGTCGGAGTCAACGGAT	CCTGGAAGATGGTGATGGG

Supplementary Table S2.xls

Differentially expressed genes. Gene list of differentially expressed genes between $CD8^+$ T_M and T_N cells at 0 hr, 4 hr and 24 hr following anti-CD3/CD28 stimulation.
